# What is primary eye health care?

**Published:** 2022-03-01

**Authors:** Clare Gilbert, Hannah Faal, Luke Allen, Matthew Burton

**Affiliations:** 1 Professor of International Eye Health: International Centre for Eye Health, London School of Hygiene and Tropical Medicine, London, UK.; 2 Adjunct Professor of International Eye health: University of Calabar Teaching Hospital, Calabar, Nigeria.; 3 Clinical Research Fellow: International Centre for Eye Health, London School of Hygiene and Tropical Medicine, London, UK.; 4 Director: International Centre for Eye Health, London School of Hygiene and Tropical Medicine, London, UK.

**Primary eye health care – just like primary health care – must include not only the provision of health care services, but also supportive multisectoral policies and action, and the empowerment of community members to take care of their eyes.**

**Figure:**
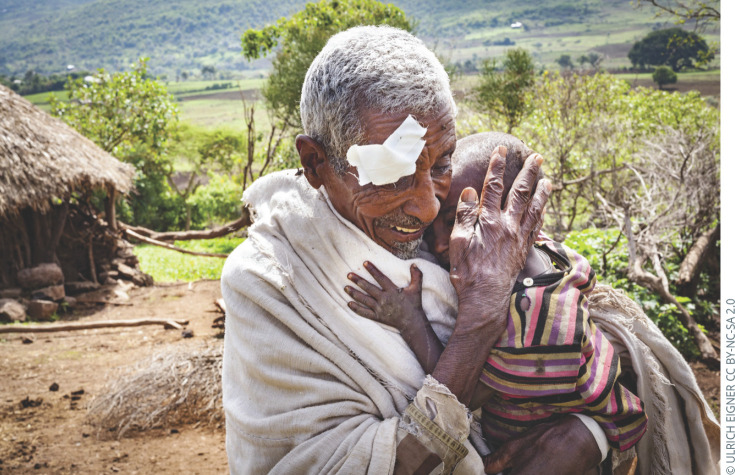
Primary eye health care has the potential to make sight-restoring surgey available to people in the remotest of communities. **BURKINA FASO**

Many of you will have heard of the term primary eye care and will understand it to mean basic eye care delivered at the first point of contact with the health service, usually by health care workers in community or primary level health facilities. In this issue, we argue that primary eye care should be seen as an integral part of **primary health care**, as defined by the World Health Organization (see panel). This term encompasses not only the service provision normally associated with the term ‘primary eye care’, but also the two health promotion components of primary health care: multisectoral policy and action, and empowered people and communities. To emphasise this distinction, we have used the term ‘primary eye **health** care’ throughout this issue of the *Community Eye Health Journal*.

**The World Health Organization definition of primary health care**

The World Health Organization (WHO), and other bodies, are increasingly recognising and advocating for better primary health care to improve population health. WHO defines primary health care as a “whole-of-society approach to health that aims to maximise the level and distribution of health and well-being through three components:

- Primary care and essential public health functions as the core of integrated health services
- Multisectoral policy and action
- Empowered people and communities.”

Each of these components contains several elements which need to be explained.

In component a, **primary care** refers to clinical services delivered by health care professionals working in the community. In high-income countries, this care may be provided by family physicians; in low-income countries general nurses or clinical officers provide these services in primary health clinics. This means that the type and level of care delivered is very context specific. **Essential public health functions and action** can include a range of activities, such as screening for disease or risk factors for disease such as hypertension, and specific preventive measures such as immunisation. **Integrated services** means that primary care is the essential, broad base of health care provision for a population, with good referral mechanisms from primary to secondary level.

Component b, **multisectoral policy and action**, refers to the importance of policies in non-health areas that influence health outcomes. This may involve multiple aspects of life, such as housing, transport, town planning, sanitation, clean air, etc., as well as specific policies which strengthen primary health care. This element is included because it is recognised that many underlying factors increase the risk of illness, such as overcrowding, air pollution, unclean water, and poor sanitation, as well as lack of public transport to access care. Policies are needed to ensure that these underlying factors are addressed and reduced. To be effective, these policies need to be adequately financed and acted upon.

Component c, **empowered people and communities**, means that people know what causes disease, what to do to remain healthy, where to go when they become sick, and how to be inclusive of those irreversibly visually disabled. They can also be told about the potential harm of using traditional or home remedies. They would then have the knowledge (health literacy) and ability (agency) to bring about the changes required.

Activities to improve health literacy, create a healthy environment, and encourage government policies that support health (the activities briefly described in b and c above) are known as **health promotion**, and we have included an article on eye health promotion in this issue (bit.ly/DeliverEye).

**Figure 1:**
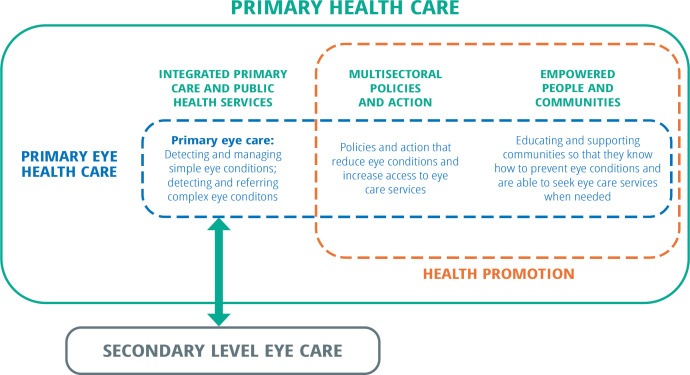
Primary eye health care (blue) is a component of primary health care (green) and consists of integrated primary care and public health services, plus health promotion (orange), which includes multisectoral policies and action as well as empowered people and communities.

## What is primary eye health care?

Referring to the three components of primary health care as defined by WHO (see panel), primary eye health care includes:

- **Primary care and essential public health functions** for eye care that are delivered by health care professionals working in facilities in the community. The type and level of eye care provided depends on the local context.
- **Multisectoral policy and action.** This refers to policies which reduce risk factors for eye conditions, such as good water supplies and sanitation, or which improve access to eye care, such as better public transport.
- **Empowered people and communities.** People know how to prevent eye conditions and are able to seek eye care services when needed.

Together, components b (multisectoral policy and action) and c (empowered people and communities), are known as **health promotion**. Read more in the health promotion article in this issue: bit.ly/DeliverEye.

## Who can deliver primary eye health care?

What eye care is delivered at primary level varies from country to country, and even within countries, depending on the resources available. Some countries have eye care personnel such as optometrists or refractionists who work in the community or in governmental, private, or non-governmental facilities (such as vision centres) where they play a vital role in providing primary eye care. In other countries, primary care facilities are staffed by a range of health professionals who may not have specific eye care training. These may include medical doctors, but more care is provided by allied health professionals such as clinical officers, nurses, and midwives, who can be trained and supported to provide basic eye care.

Given that such a high proportion of all eye problems are simple to diagnose and manage, it makes sense for this work to be carried out at primary health care level, leaving highly trained eye specialists – who usually work in secondary or tertiary level hospitals – to focus on complex eye problems.

## Who can deliver eye health promotion?

The majority of primary care clinics have health professionals attached to them whose main focus of work is in the community. They include extension or community health workers, nurse midwives, and health visitors, to name a few. These professionals are well placed to carry out health promotion activities, as detailed in the article on health promotion in this issue (bit.ly/DeliverEye).

## How can the availability of primary eye health care be improved?

Eye care professionals alone will never be able to reach everyone in a population, including marginalised groups such as those with disabilities. This means that the integration of eye health into primary health care is essential. In most countries, the majority of the population live within 10 kilometres of a primary care facility. If staff members working in, or attached to, these facilities were adequately trained, and provided with the equipment, medication, educational materials, and any other resources they need, then there is the potential for even the most remote communities to have access to primary eye health care.

There are two opportunities for integration of eye care: first, integration into primary health care provided to people of all ages, and second, integration of eye care into services which are specifically for young children. In this issue of the journal, one article makes the case for including eye care in the training curriculum of staff providing primary health care for children (bit.ly/childPEHC), and the other is an example of where this has been successfully achieved, in Bangladesh (bit.ly/banPEHC).

Primary eye health care, when it functions well, has the potential to bring about enormous change, leading to an increase in the number of people with eye conditions reaching eye care services, and a reduction in the outpatient load of secondary centres that are full of people who have simple eye conditions that could have been managed at the primary level. This issue of the *Community Eye Health Journal* contains several case studies which illustrate what can be achieved.

**Glossary of terms**

**Primary health care.** A whole-of-society approach to health that aims to maximise the level and distribution of health and well-being through primary care and essential public health functions as the core of integrated health services, alongside two health promotion measures: creating multisectoral policies and actions, and empowering people and communities.^1^

**Primary care.** Clinical services delivered by health care personnel working in community or primary care facilities. The World Health Organization defines it as a component of primary health care that supports first-contact, accessible, continued, comprehensive, and coordinated patient-focused care.^2^

**Primary eye health care.** This is, essentially, **primary health care** as it applies to eye health. Like **primary health care**, it includes **primary eye care** (see below) and essential public health functions such as screening, vaccination, or micronutrient supplementation. It also includes two broader eye health promotion measures: policies and action that reduce eye conditions and increase access to eye services, and educating and supporting communities so that they know how to prevent eye conditions and are able to seek eye care services when needed.

**Primary eye care.** Clinical eye care services delivered by health care personnel working in, or attached to, community or primary care facilities. It involves detecting and managing simple eye conditions; and detecting and referring patients with complex eye conditions to secondary level.
